# New cut-off points of PHQ-9 and its variants, in Costa Rica: a nationwide observational study

**DOI:** 10.1038/s41598-023-41560-0

**Published:** 2023-08-31

**Authors:** Armando González-Sánchez, Raúl Ortega-Moreno, Greibin Villegas-Barahona, Eva Carazo-Vargas, Harold Arias-LeClaire, Purificación Vicente-Galindo

**Affiliations:** 1 Department of Statistics and Operative Research, University of Salamanca, Campus Miguel de Unamuno. C/ Alfonso X El Sabio, S/N, 37007 Salamanca, Spain; 2 Instituto de Investigación Biomédica de Salamanca (IBSAL), Hospital Virgen de la Vega, 10ª Planta, Paseo de San Vicente, 58-182, 37007 Salamanca, Spain; 3 Psychology School, National University, Costa Rica (UNA), Heredia, Costa Rica; 4 Distance State University, Costa Rica (UNED), San Jose, Costa Rica

**Keywords:** Human behaviour, Scientific data

## Abstract

The PHQ-9 questionnaire is a screening test worldwide used to measure depression. But it cannot be used in Costa Rica, due to the fact that it has not previously been validated for its population. The present study aims to show the validation of the PHQ-9 questionnaire and its variants (PHQ-2, PHQ-4, PHQ-8) in a population sample of adults residing in Costa Rica. A sample was collected (n = 1162) using a self-administered questionnaire. Confirmatory Factor Analysis (CFA), Receiver Operating Characteristic (ROC) curve, and Multiple Group Confirmatory Factor Analysis (MGCFA) were tested. One factor was found that explained 73.33% of the variance with excellent internal consistency (α = 0.928). Goodness-of-fit measures were adequate (RMSEA = 0.107; CFI = 0.948), as was diagnostic power at a cut-off of 10 (78.60 for Sensitivity and 27.95 for 1-Specificity). External validation indices were good (r = 0.843 with GAD-7, r = − 0.647 with RS14, and r = 0.301 with FCV19S), and the model showed invariance by sex (∆χ^2^ = 27.90; df = 27; *p* < 0.001). Additionally, new cut-off points were proposed for PHQ-9 and its variants for Costa Rican male, female, and general populations. The PHQ-9 and its variants (PHQ-2, 4, and 8) are valid tools for detecting depression (and anxiety for PHQ-4) in Costa Rican population. In addition, new cut-off points differentiated by sex are proposed.

## Introduction

Depression is a deterioration of a person’s mental health that implies a lower ability of the individual to function, characterized by sadness, emptiness, or irritability^1^. By influencing the ability to perform tasks, the depression can produce a reduction in productivity and impairment that implies disability^2,3^. The age of onset for depressive disorders is between 30 and 35 years old^4^. Among the factors involved in the tendency to suffer from depression are job instability and low social support^5–7^.

Depressive symptoms constitute one of the main mental health problems; thus, most psychiatric care is devoted to treating depression^8^. Worldwide, it is estimated that 12.26% of the population suffers from mental disorders, with depression at 3.44%; in Central America, the prevalence of depressive disorders stands at 3.19%^9^. In Costa Rica, the prevalence of concurrent symptoms with major depressive disorder was 9.5% at pre-pandemic dates due to COVID-19 (n = 797), while during the pandemic it was 61.0% (n = 6786)^7^. In addition, it has a rate of 7.6 (95% CI 5.29–10.53) deaths by suicide per 100,000 inhabitants in the country, ranking above most countries in the Americas^10^. Although one of the characteristics of depression is suicidal ideation, it is difficult to predict imminent suicide^11^.

There are sexual differences in the comorbidity of mental disorders, since women tend to internalize disorders, while men tend to develop external disorders such as substance misuse^6^. As in the rest of the world, in Costa Rica women have a higher prevalence of depression than men^7,12^, although the reasons for these differences are still being studied^4,13^.

This has consequences to the economy, due to inability to carry out work activities, in the quality of life due to the disability it produces, loss of life, the reduction of educational opportunities, work and social connections, affecting future generations^3,14,15^. In Costa Rica, 8.0% of disability is due to exclusively depressive disorders, excluding substance-related disorders, self-harm, and suicide; mainly affecting working-age people^3^. This implies that mental health depends in part on the social and economic conditions in which the person finds him- or herself.

Being a treatable and preventable disease, its early identification avoids health complications. Therefore, prevention, early detection, and treatment efforts are essential^15^. With adequate detection of depression, $11,134 per year could be saved per woman and $34,065 per man^15^. Thus, routine detection of depression in populations is cost-effective regardless of gender or age^15^. Population screenings are effective both through self-administered surveys and during the COVID-19 pandemic^16^.

The benefits of detecting depression early can only be achieved with proper studies, since making erroneous diagnoses not only makes detection more difficult but can also lead to great emotional distress, worse prognosis, and discrimination by social characteristics or group belonging^17–20^.

Therefore, it would be necessary to consider the importance of screening the population properly. If a tool is used in circumstances other than those for which it was designed, such as a different culture or language, adaptations must be made so that the tools are equivalent^21^. The equivalence of them in populations is closely linked to validity, especially when administered across cultures^22^. Conceptual equivalence (the meaning attributed to a given concept), items (guaranteeing relevance, clarity and relevance), semantics (cross-cultural meaning), operation (administration, qualification and interpretation under the same conditions for quality assessment), measurement (referring to identical psychometric properties between cultures) and functional equivalence (fulfillment of test purpose: adequate measurement of construct while respecting group independence) are all important considerations^23^.

The PHQ-9 is a test used for assessing the severity of depression in adults. It can be completed within 5 min and is self-administered. It can be used for monitoring the evolution of depression in response to treatment^24,25^. It can be applied to several patients and have results in a short time. It is often used in primary care to diagnose depression and its severity quickly and efficiently^26^. It has been studied for its high relationship with the anxiety test GAD-7^27,28^, and Fear of Covid-19S^29^, and indirect relationship with resilience tested by RS14^30,31^.

PHQ-9 has been translated into many different languages^32–35^. It has been validated in Latin American countries such as Peru^36,37^ or Chile^38,39^, and has been widely used as a tool for identifying symptoms associated with depression during the COVID-19 pandemic throughout the world^40–43^, However, as of the date of the presentation of this document, there is no reference to a validation in Costa Rica.

There have only been a few studies of depression in Costa Rica, offering prevalence data that is not close to other populations^44^. In this way, prevalence studies place depression with very different values: 1.08%^45^, 12.6^46^ and 27.3^7^. Likewise, in women it manifests itself with a ratio of 3 to 1. Culturally, men do not express their feelings and it is only after 30 years of age that the first diagnoses of depression are given^47^.

The main goal of this article is to evaluate the psychometric properties of the Spanish version of the PHQ-9 and its variants: PHQ-8, PHQ-4, and PHQ-2 questionnaires, in a sample of Costa Rican adults by testing the factorial structure^48^. We expect it to have a unifactorial structure, as in the original validation of the tool in Spanish^49^. The second objective is to assess the cross-sectional multigroup invariance (MGCFA) (female versus male participants) of the PHQ-9 test and its variants.

To do this, the descriptive statistics of the tests will be verified, their psychometric properties evaluated, new cut-off points established for both the general population and differentiated by sex, MGCFA multigroup invariance verified, and relationships between the tests established through multivariate analysis.

## Methods

### Study population

Costa Rica is the Latin American country listed as the happiest^50^. The country lacks robust health coverage and mental health research is not advanced; This implies that data on the characteristics of depression are unknown^51^. This circumstance is more accentuated in rural areas where mental health professionals are scarcer^51^. Additionally, addresses are often described in relation to local landmarks rather than street names or house numbers, leading to complications in providing mental healthcare.

Costa Rica population has a total of 84.7% of internet access (87.0% urban and 78.7% rural), also 96% have phones, 47% have computer, and 16.7% have tablet^52^. Costa Rica has a low level of upper secondary and post-secondary educational level (18%) and the lowest percentage of doctoral achievement 0.1% of the OECD countries^53^.

Its ethnic distribution is made up of: Caucasians, who constitute 83.6%, mixed race 6.72%, South Amer Indians 2.42%, the rest are Undeclared or Others^54^.

Costa Rican Spanish recognizes twelve linguistic features spread across five large regions, forming internal sociolinguistic differences^55^, there being a complex compendium of unique social characteristics such as *gypsyisms* such as the *achará*^56^, making it difficult to understand and express the language among other cultures. Where other versions of Spanish are spoken^57^, such as Spanish or Mexican the questionnaire was validated previously.

The inclusion criterias were adults residing in Costa Rica at the time of evaluation, ability to read, and understand Spanish, and having expressed their explicit consent to participate in the study.

A total of 14,702 responses were obtained. Of these, 13,506 responses had some missing data and were eliminated. To facilitate responses, completing the survey was not made mandatory. Data such as age and sex, or incomplete responses to the tool were used as criteria for eliminating responses. Given that we had sufficient data, a decision was made to perform data cleaning of this magnitude. An additional 34 responses were removed due to being completed too fast. Our sample consisted of 1162 completed surveys. The age, gender, civil status, education level, province, and employment status of each participant were recorded (Table 1).

**Table 1:** Sociodemographic characteristics of the sample.

Sociodemographic variable	Response option	Value (n. %)
Gender	Male	247 (21.3)
	Female	915 (78.7)
Civil status	Single	561 (48.3)
	Married	361 (31.1)
	Coupled	116 (10.0)
	Divorced	94 (8.1)
	Separated	19 (1.6)
	Widowed	9 (0.8)
	Missing	2 (0.2)
Education level	Non universitarian	598 (51.5)
	Universitarian	378 (32.5)
	Master	167 (14.4)
	PhD	19 (1.6)
Province	San José	459 (39.5)
	Alajuela	176 (15.1)
	Cartago	205 (17.6)
	Heredia	149 (12.8)
	Guanacaste	94 (8.1)
	Puntarenas	42 (3.6)
	Limón	37 (3.2)
Labor condition	Employed	651 (56.0)
	Unemployed	105 (9.0)
	Others	406 (34.9)

In a previous analysis, a minimum sample size of 1100 subjects was required to calculate structural equation models^58^.

### Instruments

The use of the PHQ-9 during the pandemic allows for reaching adequate conclusions^59^. The PHQ-9 was administered, and is used to detect symptomatology concurrent with depression by screening, which has excellent psychometric properties, both its internal consistency (α = 0.89) and its test–retest reliability (ICC = 0.84)^26,60^. Based on Classical Test Theory, its items have scores ranging from 0 to 3. The PHQ-9 scale has total scores ranging from 0 to 27 and is obtained by adding the score of each item. The test correction has cut-off points of 5, 10, 15, and 20, resulting in the following classification: minimal depression (0–4); mild depression (5–9); moderate depression (10–14); moderate to severe depression (15–19); and severe depression (20–27). The PHQ-9 test consists of nine main items plus an additional three items that ask if you have experienced discomfort due to any of the problems mentioned in the main items. These additional items refer to the difficulty caused by these problems in three contexts: work, household tasks, or social relations. The last three items have response options ranging from 0 (no difficulty) to 3 (extreme difficulty).

For this research, the team made minor modifications taking from example various versions of the PHQ-9 test. We selected items from different versions of the test that had already been validated in other Spanish-speaking countries. These cultural modifications were made to improve comprehension while preserving the original meaning of the items (see Supplementary material).

The PHQ-9 has several variants. The PHQ-2 version is considered within the PHQ-9 as it corresponds to its first two items, forming a reduced version of the main version. Additionally, these two items are considered the core criterion for depressive disorder. Its score range goes from 0 to 6, with scores greater than or equal to 3 indicating depression^61–64^. The PHQ-8 version includes all items of the PHQ-9 except for the ninth item related to suicide and indicates current depression at a cut-off point ≥ 10^65^. The PHQ-4 version consists of the first two items of the PHQ-9 combined with the first two items of the GAD-7 (General Anxiety Disorder-7), thus examining both depression and anxiety^66^.

For external validity, we used tests to assess anxiety, resilience, and fear of Covid. The GAD-7 is a screening scale for measuring generalized anxiety disorder^67^. It has strong internal consistency (α = 0.94) and good test–retest reliability (ICC = 0.83) and uses seven items with response options ranging from 0 to 3. The total score of the test results from the sum of its items, with minimum and maximum scores ranging from 0 to 21. The thresholds proposed in its original scale are: minimal anxiety (0–4), mild anxiety (5–9), moderate anxiety (10–14), and severe anxiety (15–21). The GAD-2 questionnaire is included in the GAD-7 and corresponds to its first two items. A score ≥ 3 indicates clinically relevant anxiety disorder^62,67–69^. The RS-14 Resilience test measures an individual’s degree of adaptation to adverse situations. This scale consists of 14 items with theoretical scores ranging from 98 to 14, indicating different levels of resilience according to this score range: very high resilience (98–82), high resilience (81–64), fair (63–49), low resilience (48–31), and very low resilience (30–14)^70^. The Fear of Covid-19 Scale measures fear of Coronavirus disease (FCV-19S). With theoretical scores ranging from 7 to 35^71,72^, it indicates a direct relationship between fear of illness and test score.

Finally, as an external criterion, questions about current depression and anxiety were included in the tool: suffering from depression; suffering from anxiety; taking medication in the last 30 days for depression, sleep, or anxiety; or being in psychiatric or psychological treatment in the last 30 days.

Informed consent was obtained from all participants.

### Procedure

A self-administered survey hosted on an online survey platform was used (LimeSuvey). The access was made through a link. The survey was answered by adults from the general population of Costa Rica for 8 months (between March 22nd and September 22nd, 2021), during the second year of the pandemic in the country. The population just needed internet and a device for answering the survey, as it can be done using a mobile device, tablet, or computer. Participation in this study was made from the Ministry of Health of Costa Rica, Distance Education University of Costa Rica (UNED), the National University (UNA), and the Costa Rican Social Security Fund (CCSS). The institutions requested participation through their institutional websites, and their social networks: Facebook and Twitter.

Four attentional check items were distributed throughout the questionnaire: “If you are reading this, please select the following option: totally agree/agree/never/always”.

Participation was assured to be anonymous, confidential, voluntary, and participants could withdraw from the survey at any time. Additionally, information on national psychological care and resources was available at the end of the survey.

All subjects gave their informed consent for inclusion before they participated in the study. The study was conducted in accordance with the Declaration of Helsinki, and the protocol was approved by the Ethics Committee of the Ministry of Health through the National Health Research Council (CONIS) under Agreement No. 22 of the ordinary session No. 38 of August 26, 2020, with the number: CONIS-242-2020 confirming that all research was performed in accordance with relevant guidelines or regulations. This study was not preregistered.

### Data analysis

First, descriptive statistics were obtained for frequency, means, standard deviation, asymmetry, and kurtosis using the statistical program SPSS v.25^73^. To estimate concurrent validity, Spearman’s Rho and Pearson’s correlations were used.

The FACTOR v.12.01.02 program^74^ was used for internal validity analyses, descriptives, frequencies and item properties. Factor analysis was performed following recommended standards^75^. Matrix analysis was conducted through polychoric correlation with the Minimum Rank Factor Analysis factor model (MRFA)^76^. To determine the Kaiser–Meyer–Olkin index (KMO), the entire sample was used as two random subsamples with the Solomon method^77^. In the analysis presented below, explained variance is based on eigenvalues. Additionally, the Measure of Sampling Adequacy (MSA) statistic is offered which advises eliminating items with values less than 0.50^74^. For internal consistency, McDonald’s Omega (Oω) statistic was used under the Omega macro for SPSS beta 0.2 package^78^.

To calculate sex invariance in the model, Multi-Group Confirmatory Factor Analysis (MGCFA) was used and executed using AMOS^79^. Changes in χ^2^ were reported with their degrees of freedom and *p*-values. The parameters AIC, GFI and RMSEA were reported to demonstrate goodness-of-fit to a single model factor structure for PHQ-9 test.

To calculate optimal cohort points for depression, being in psychiatric or psychological treatment for depression was an additional question, this determines robustly the two different groups: one with a depressive trait and another without it. Similarly, to establish cut-off points for PHQ-4 test, criteria such as suffering from depression while also suffering from anxiety were used. The Youden’s J index and Closest-top-left criteria on ROC curve were used; when these indexes did not coincide, Youden’s J statistic was chosen^80^. Sensitivity, specificity, and area under ROC curve are also presented. Cut-off points for PHQ-8 were established based on its original version while respecting severity length from optimal shear point^65^.

Finally, to calculate new cut-off points indicating severity of depression for each test, corresponding bases were added to cut-off points obtained using Youden J indices and Closest top left on ROC curve with respect to ranges composing each court to establish severity of depressive symptoms.

A confidence level of 0.05 was marked as significant for all analyses including confidence intervals (CI) at 95%.

## Results

### Participants

A total of 1162 people participated in study with a mean age of 35.52 years old (SD = 12.18) ranging from 15 to 78 years old (Table 1).

### Psychometric properties

The mean and standard deviation of PHQ-2, PHQ-4, PHQ-8, and PHQ-9 scales were 2.38 (1.94), 4.97 (3.65), 9.33 (6.90) and 9.80 (7.43) respectively, with scores reach-ing theoretical range of tests.

Internal consistency of items was high, with lower scores in item 9 of depression (Table 2). Among different scales, high correlations were observed, positive for depression and fear of COVID-19 and negative for resilience (Table 2).

**Table 2:** Correlation table. Spearman's Rho bivariate correlations down, Pearson’s up.

Item number and scale	1	2	3	4	5	6	7	8	9	10	11	12	13	14	15	16	17	18	19	20
1	1	0.744**	0.604**	0.657**	0.637**	0.675**	0.634**	0.532**	0.471**	0.613**	0.558**	0.584**	.931**	0.838**	0.832**	0.651**	0.678**	0.849**	0.211**	− 0.532**
2	0.752**	1	0.654**	0.672**	0.621**	0.760**	0.620**	0.572**	0.581**	0.613**	0.551**	0.635**	0.937**	0.865**	0.870**	0.702**	0.742**	0.880**	0.257**	− 0.563**
3	0.609**	0.658**	1	0.702**	0.647**	0.593**	0.558**	0.509**	0.434**	0.533**	0.509**	0.532**	0.674**	0.812**	0.803**	0.636**	0.668**	0.705**	0.268**	− 0.435**
4	0.662**	0.676**	0.697**	1	0.675**	0.626**	0.567**	0.502**	0.404**	0.581**	0.569**	0.556**	0.711**	0.829**	0.816**	0.700**	0.729**	0.760**	0.291**	− 0.457**
5	0.635**	0.614**	0.636**	0.670**	1	0.649**	0.612**	0.546**	0.446**	0.557**	0.555**	0.539**	0.673**	0.828**	0.820**	0.645**	0.679**	0.710**	0.296**	− 0.472**
6	0.677**	0.756**	0.586**	0.619**	0.633**	1	0.614**	0.545**	0.543**	0.565**	0.541**	0.592**	0.769**	0.840**	0.842**	0.645**	0.686**	0.760**	0.252**	− 0.567**
7	0.614**	0.605**	0.546**	0.561**	0.596**	0.596**	1	0.618**	0.492**	0.580**	0.545**	0.571**	0.671**	0.794**	0.794**	0.599**	0.653**	0.683**	0.262**	− 0.474**
8	0.512**	0.555**	0.493**	0.492**	0.523**	0.519**	0.588**	1	0.457**	0.523**	0.507**	0.515**	0.592**	0.727**	0.728**	0.556**	0.629**	0.617**	0.224**	− 0.449**
9	0.460**	0.552**	0.416**	0.403**	0.424**	0.534**	0.470**	0.427**	1	0.447**	0.394**	0.472**	0.565**	0.585**	0.659**	0.463**	0.504**	0.553**	0.087**	− 0.501**
10	0.622**	0.627**	0.537**	0.583**	0.558**	0.571**	0.585**	0.521**	0.437**	1	0.715**	0.652**	0.656**	0.697**	0.699**	0.642**	0.675**	0.699**	0.292**	− 0.514**
11	0.568**	0.567**	0.516**	0.574**	0.553**	0.543**	0.543**	0.503**	0.384**	0.713**	1	0.645**	0.593**	0.662**	0.660**	0.601**	0.638**	0.643**	0.323**	− 0.473**
12	0.592**	0.641**	0.542**	0.564**	0.539**	0.590**	0.562**	0.499**	0.449**	0.662**	0.655**	1	0.653**	0.692**	0.696**	0.612**	0.658**	0.681**	0.294**	− 0.523**
13-PHQ2	0.928**	0.939**	0.678**	0.715**	0.666**	0.765**	0.647**	0.568**	0.543**	0.663**	0.600**	0.656**	1	0.912**	0.911**	0.725**	0.761**	0.926**	0.251**	− 0.587**
14-PHQ8	0.838**	0.864**	0.816**	0.843**	0.821**	0.828**	0.764**	0.680**	0.551**	0.705**	0.667**	0.697**	0.910**	1	0.996**	0.786**	0.836**	0.913**	0.316**	− 0.604**
15-PHQ9	0.836**	0.868**	0.811**	0.836**	0.816**	0.831**	0.764**	0.678**	0.601**	0.707**	0.667**	0.700**	0.911**	0.998**	1	0.783**	0.834**	0.911**	0.303**	− 0.618**
16-GAD2	0.661**	0.709**	0.644**	0.704**	0.640**	0.644**	0.593**	0.539**	0.449**	0.648**	0.609**	0.623**	0.733**	0.796**	0.796**	1	0.938**	0.931**	0.396**	− 0.500**
17-GAD7	0.687**	0.748**	0.675**	0.738**	0.674**	0.685**	0.638**	0.605**	0.472**	0.683**	0.647**	0.671**	0.767**	0.843**	0.843**	0.937**	1	0.916**	0.432**	− 0.524**
18-PHQ4	0.851**	0.881**	0.711**	0.764**	0.703**	0.753**	0.664**	0.593**	0.530**	0.702**	0.647**	0.685**	0.927**	0.916**	0.916**	0.931**	0.917**	1	0.349**	− 0.584**
19-FCV19S	0.211**	0.257**	0.265**	0.282**	0.289**	0.254**	0.254**	0.210**	0.073*	0.272**	0.314**	0.286**	0.246**	0.310**	0.301**	0.387**	0.418**	0.338**	1	− 0.184**
20-RS14	− 0.575**	− 0.600**	− 0.462**	− 0.486**	− 0.493**	− 0.601**	− 0.490**	− 0.448**	− 0.508**	− 0.548**	− 0.503**	− 0.554**	− 0.625**	− 0.636**	− 0.647**	− 0.534**	− 0.560**	− 0.620**	− 0.199**	1

Items 10, 11, and 12 correspond to the impact of depression in work, household, and social areas, respectively. All relationships were highly significant (*p* < 0.001), except for the relationship between the total FCV-19S score and item 9 of the PHQ-9, which was significant at 5% (*p < 0.05; **p < 0.01).

Eliminating any item did not increase the internal consistency of the questionnaire (Table 3). The suicide item represents an extreme condition of depression and low scores. It forms part of the general dimension of depression and is recommended to remain on the scale to assess depression. Item 4 (loss of energy) is considered the easiest item to answer even though items 1 and 2 make up the core of major depressive syndrome.

**Table 3:** Descriptives, item properties and frequencies. PHQ-9.

Psychometrics	No. Item									
Dimension	Parameter	1	2	3	4	5	6	7	8	9
Item properties	Fixed correlation item-total	0.78	0.83	0.74	0.76	0.76	0.79	0.74	0.66	0.59
	α if item deleted	0.92	0.91	0.92	0.92	0.92	0.92	0.92	0.93	0.93
	Load factors	0.84	0.87	0.80	0.82	0.82	0.84	0.80	0.73	0.66
	QIM	3	3	3	3	3	3	3	2	2
	RDI	0.40	0.39	0.45	0.54	0.42	0.37	0.31	0.21	0.15
	Normed MSA	0.94	0.90	0.95	0.94	0.94	0.95	0.95	0.95	0.94
Descriptives	Mean	1.20	1.18	1.36	1.62	1.27	1.12	0.94	0.63	0.46
	SD	1.01	1.06	1.13	1.06	1.10	1.14	1.00	0.92	0.86
	CI 95%	(1.12–1.28)	(1.10–1.26)	(1.27–1.44)	(1.54–1.70)	(1.19–1.36)	(1.04–1.21)	(0.87–1.02)	(0.56–0.70)	(0.40–0.52)
	Skewness	0.48	0.51	0.29	0.06	0.37	0.53	0.78	1.37	1.88
	Kurtosis	− 0.85	− 0.96	− 1.31	− 1.30	− 1.19	− 1.15	− 0.51	0.82	2.48
	Loading weights by ULS	0.87	0.93	0.83	0.87	0.84	0.88	0.83	0.79	0.76
	Communality	0.70	0.76	0.64	0.66	0.67	0.71	0.63	0.53	0.68
Answer frequencies	Not at all	324	365	323	172	351	468	486	696	838
	Several days	459	423	386	441	381	303	384	283	186
	More than half the days	201	170	165	204	190	173	162	95	65
	Nearly every day	178	204	288	345	240	218	130	88	73

The PHQ-9 test had a determinant close to zero (0.002) and obtained a similar KMO measure for both subsamples (0.937 and 0.933) and for the complete sample (0.996). A single factor was extracted that explained 73.33% of the variance. The eigenvalue of its next component did not reach unity (0.542).

The respective indices were obtained for different versions of PHQ-9 to calculate their internal consistency and factorial structure (Table 4). McDonald’s Omega could not be calculated for PHQ-2 because it only has two items; thus, a reduction in dimensions was not possible. However, results are presented for all cases where a single factor was extracted.

**Table 4:** Psychometric properties of the different versions of the PHQ-9.

Dimension	Parameter	PHQ2	PHQ4	PHQ8	PHQ9
Psychometrics	Explained variance (1 factor)	91.47	76.84	75.43	73.39
	KMO	0.5	0.780	0.930	0.930
	Bartlett	1350.2 NS	3110.5**	8881.6**	9957.6**
	Alpha (α)	0.853	0.899	0.929	0.928
	McDonald’s Omega (Oω)	NA	0.898	0.930	0.931
	Items removed by MSA^a^	NA	NA	NA	NA
Goodness-of-fit (1 factor)	χ^2^_gf_	Undefined	45.75_2_ (< 0.001)	309.91_20_ (< 0.001)	386.71_27_ (< 0.001)
	AIC^b^	–	61.75	341.91	422.71
	GFI	–	0.915	0.936	0.927
	RMSEA	0.898	0.298	0.112	0.107
	∆χ^2^_gf_ by sex (*p*-value)	–	261.22_4_ (< 0.001)	329.03_40_ (< 0.001)	27,90_27_ (< 0.001)

^a^MSA, Measure of Sampling Adequacy. ^b^AIC were calculated using the default model. *p-value > 0.05. **p-value > 0.01.

### Cut-off-points

**Figure 1:**
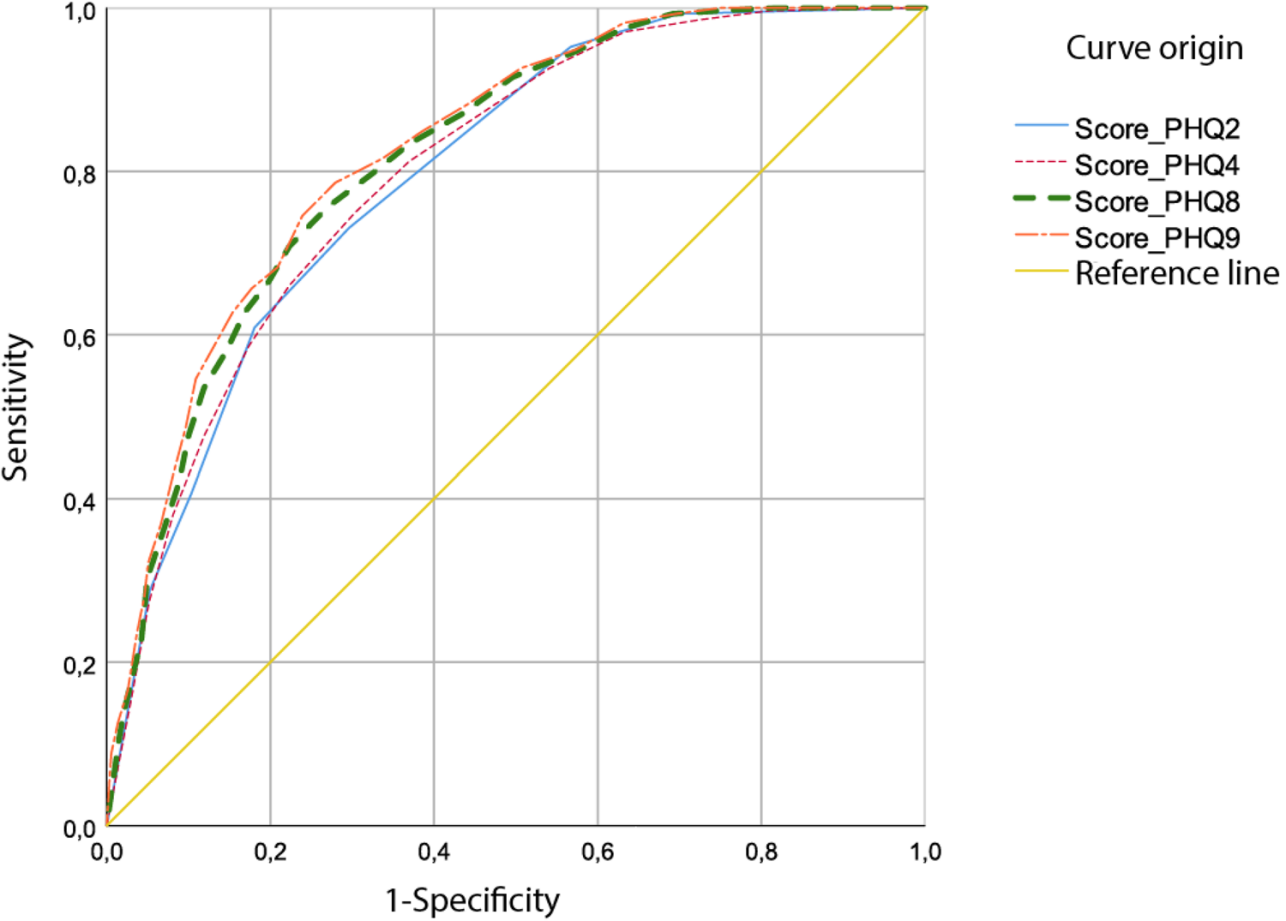
ROC curve for the different versions of PHQ regarding the diagnosis of depression.

As shown in Fig. 1, different versions of PHQ-9 function similarly for depression prior to diagnosis criteria. Receiver Operating Characteristic (ROC) curves can be ordered by the number of items included in each questionnaire version.

When analyzing the segment in the upper left corner (Closest-top-left) on the ROC curve, tests with more items have better specificity and sensitivity indices; however, these differences are slight (Table 5).

**Table 5:** Selected sensitivity and specificity based on perceived depression.

Type	Version	Score	J Youden´s index	Closest top left	Sensitivity (%)	1-Specificity (%)
General	PHQ2	2.5	0.434	0.400	73.06	29.63
	PHQ4	5.5	0.446	0.393	74.90	30.30
	PHQ8	10.5	0.486	0.364	75.65	27.05
	PHQ9	*10.5*	*0.507*	0.352	78.60	27.95
		11.5	0.506	*0.349*	74.54	23.91
Male	PHQ2	2.5	0.472	0.374	75.00	27.80
	PHQ4	4.5	0.485	0.371	79.20	30.70
	PHQ8	8.5	0.516	0.342	76.70	25.10
	PHQ9	10.5	0.551	0.328	71.70	16.60
Female	PHQ2	2.5	0.424	0.408	72.51	30.11
	PHQ4	5.5	0.450	0.394	76.80	31.80
	PHQ8	11.5	0.496	0.357	73.90	24.30
	PHQ9	11.5	0.512	0.346	77.30	26.10

Discrepant values were obtained for PHQ-9 test for indices indicating optimal cut-off point. If Youden’s J prevails over Closest Top Left, optimal cut-off point for PHQ-9 is ≥ 10.

The italics indicates the selected index and its associate score: the highest J Youden's Index and the smallest Closest top left index.

Table 5 shows different cut-off points of PHQ versions for both the general population and the population according to sex. Optimal thresholds for the general population coincide with PHQ-2 but are disaggregated in the rest. Consequently, the optimal cohort points for men are 1 or 2 points lower than for women, depending on the version administered. In PHQ-8 and PHQ-9 for women, the optimal cut-off score is 11 points. On the other hand, for males, the optimal score is 8 and 10 points, respectively. Below is Table 6 summarizing different thresholds found as well as the severity of depression according to different segments.

**Table 6:** Cut-off points for the severity of depressive symptoms of the PHQ-9 versions in the Costa Rican general population, its original measure and new cut-off points split by sex.

Group	Version	Non major depressive disorder	Major depressive disorder	None to minimal	Mild	Moderate	Moderately severe	Severe
Original	PHQ2	0–2	3–6	–	–	–	–	–
	PHQ4	–	–	0–2	3–5	6–8	–	9–12
	PHQ8	–	–	0–4	5–9	10–14	15–19	20–24
	PHQ9	–	–	0–4	5–9	10–14	15–19	20–27
General population	PHQ2	0–2	3–6	–	–	–	–	–
	PHQ4	–	–	0–2	3–5	6–8	–	9–12
	PHQ8	–	–	0–5	6–10	11–15	16–20	21–24
	PHQ9	–	–	0–5	6–10	11–15	16–20	21–27
Male	PHQ2	0–2	3–6	–	–	–	–	–
	PHQ4	–	–	0–1	2–4	5–7	–	8–12
	PHQ8	–	–	0–3	4–8	9–13	14–18	19–24
	PHQ9	–	–	0–5	6–10	11–15	16–20	21–27
Female	PHQ2	0–2	3–6	–	–	–	–	–
	PHQ4	–	–	0–2	3–5	6–8	–	9–12
	PHQ8	–	–	0–6	7–11	12–16	17–21	22–24
	PHQ9	–	–	0–6	7–11	12–16	17–21	22–27

## Discussion

The PHQ scale demonstrates adequate internal consistency (with an α range of 0.853 to 0.929) for different PHQ versions analyzed. These values are like those found in other studies^26,60,81^. All versions studied exhibit concurrent validity with positive and high correlations for GAD-7 (ranging from 0.767 to 0.931), positive but slight for FCV-19S (ranging from 0.246 to 0.338), and negative but moderate for RS-14 (ranging from − 0.620 to − 0.647), all of which are consistent with expectations.

The PHQ-9 exhibits high inter-item correlations like those found in other studies (ranging from 0.416 to 0.756). As expected, the suicide item has lower but still significant correlations (ranging from 0.416 to 0.552)^82^.

As an indicator of internal validity, a single factor was obtained in all PHQ-9 variants^63–65^ including the PHQ-4, which also yielded a single factor despite comprising two different theoretical dimensions^66^.

The thresholds for the general population coincide with those proposed in its original version. To assess depression in men, cut-off points have been lowered for both PHQ-4 and PHQ-8. In contrast, to assess depression in women, original thresholds for PHQ-2 and PHQ-4 have been maintained; however, cut-off points have been increased for both PHQ-8 and PHQ-9. If cut-off points from this study were not applied, men would be underdiagnosed, and women would be overdiagnosed.

Since scores from PHQ-8 are assimilated to those of PHQ-9 for convenience in its original article, this alters thresholds of PHQ-8 in practice leaving less scope for extreme scores. Although new cut-off points have been established to maintain the nature of PHQ-8, it continues to have less range in the severe part of depression.

The diagnostic validity of PHQ-9 with a sensitivity of 78.60% and a specificity of 72.05% presents lower but adequate indices compared to its versions in English (88% sensitivity and 88% specificity)^26^, Spanish (87% sensitivity and 88% specificity)^35,49^, Mexico (for PHQ-2) 80.0 and 86.9^83^.

Despite having only two items, PHQ-2 can detect depression with slightly lower sensitivity and specificity values. Considering the reduction in items, it maintains excellent psychometric properties compared to its longer version. Since PHQ-4 also assesses anxiety, it was compared with the anxiety-depression construct, obtaining a ROC curve like that obtained by comparing it only with depression.

A limitation is although both gender and sex of each patient were assessed, a sample of people born with a gender other than the one identified was too small to form a representative group. Another limitation is the race or ethnicity of participants was not assessed. A large number of discarded tests could be not considered a limitation due to the sample size is being sufficient and data cleaning meeting valid criteria. An explanation for missing data is that this research was published on TV, radio, newspaper, and institutional web pages. The health minister and other relevant public figures asked the population to fill out the questionnaires. That phenomenon made most of the population enter the platform just to satisfy their curiosity. This could be a limitation to not retaining most of the participants to fill all the survey.

Although our sample is representative and heterogeneous in terms of age, sex, academic level, and marital status, we acknowledge that no formal approach was followed to ensure this. This is a cross-sectional study, so a future longitudinal follow-up could be more valuable. Convenience sampling represents a weakness in the generalization of the findings of the present study. Another sampling limitation is that in the country the devices for accessing the internet are not well distributed^52^, and because of this, the population who are without internet access couldn’t be contacted. Despite these limitations, this study provides valuable information on the usefulness of the application of the PHQ-9 within the Costa Rican population.

## Conclusions

Due to the ability to detect depression, brevity of application, and self-administered application, use of PHQ-2, PHQ-4, PHQ-8 and PHQ-9 tests are considered adequate tools for detecting depression and its severity in the general Costa Rican population. Due to the high consistency of this test, its application is considered valid in the entire Spanish-speaking community.

Proposed thresholds improve the detection of depression based on gender.

### Supplementary Information

Supplementary Information.

## Data Availability

Data are available from the correspondence author (Armando González-Sánchez. armando_gonzalez@usal.es), upon request. Data are not publicly available due to ethical issues.
