# Author Correction: New cut-off points of PHQ-9 and its variants, in Costa Rica: a nationwide observational study

**DOI:** 10.1038/s41598-024-55470-2

**Published:** 2024-03-08

**Authors:** Armando González-Sánchez, Raúl Ortega-Moreno, Greibin Villegas-Barahona, Eva Carazo-Vargas, Harold Arias-LeClaire, Purificación Vicente-Galindo

**Affiliations:** 1 Department of Statistics and Operative Research, University of Salamanca, Campus Miguel de Unamuno. C/ Alfonso X El Sabio, S/N, 37007 Salamanca, Spain; 2 Instituto de Investigación Biomédica de Salamanca (IBSAL), Hospital Virgen de la Vega, 10ª Planta, Paseo de San Vicente, 58-182, 37007 Salamanca, Spain; 3 Psychology School, National University, Costa Rica (UNA), Heredia, Costa Rica; 4 Distance State University, Costa Rica (UNED), San Jose, Costa Rica

Correction to: *Scientific Reports* 10.1038/s41598-023-41560-0, published online 31 August 2023

The Supplementary Information file published with this Article contained an error, where the Costa Rican version of the Patient Health Questionnaire-9 was omitted. The original Supplementary Information file is provided below.

The error has now been corrected in the Supplementary Information file that accompanies the original Article.

### Supplementary Information

Supplementary Information.

