# Supplementary material for: New cut-off points of PHQ-9 and its variants, in Costa Rica: a nationwide observational study
Source: Sci Rep. 2023 Aug 31;13:14295. doi: 10.1038/s41598-023-41560-0 (PMC10471633; doi:10.1038/s41598-023-41560-0)
Supplement: Supplementary file 1 — Supplementary Information. [file 41598_2023_41560_MOESM1_ESM.pdf]

PATIENT HEALTH QUESTIONNAIRE-9 (PHQ-9)

**Over the last 2 weeks, how often have you been bothered by any of the following problems?** *(Use "✔" to indicate your answer)*	**Not at all**	**Several days**	**More than half the days**	**Nearly every day**
**1.** Little interest or pleasure in doing things	0	1	2	3
**2.** Feeling down, depressed, or hopeless	0	1	2	3
**3.** Trouble falling or staying asleep, or sleeping too much	0	1	2	3
**4.** Feeling tired or having little energy	0	1	2	3
**5.** Poor appetite or overeating	0	1	2	3
**6.** Feeling bad about yourself — or that you are a failure or have let yourself or your family down	0	1	2	3
**7.** Trouble concentrating on things, such as reading the newspaper or watching television	0	1	2	3
**8.** Moving or speaking so slowly that other people could have noticed. Or the opposite — being so fidgety or restless that you have been moving around a lot more than usual	0	1	2	3
**9.** Thoughts that you would be better off dead or of hurting yourself in some way	0	1	2	3

FOR OFFICE CODING __0__ + ______ + ______ + ______

=Total Score: ______

If you ticked any problems, how difficult have these problems made it for you to do your work, take care of things at home, or get along with other people?

**Not difficult at all**	**Somewhat difficult**	**Very difficult**	**Extremely difficult**
☐	☐	☐	☐

Developed by Drs. Robert L. Spitzer, Janet B.W. Williams, Kurt Kroenke and colleagues, with an educational grant from Pfizer Inc. No permission required to reproduce, translate, display or distribute.

PHQ-4

**Over the last 2 weeks, how often have you been bothered by the following problems?** *(Use "✔" to indicate your answer)*	Not at all	Several days	More than half the days	Nearly every day
1. Feeling nervous, anxious or on edge	0	1	2	3
2. Not being able to stop or control worrying	0	1	2	3
3. Little interest or pleasure in doing things	0	1	2	3
4. Feeling down, depressed, or hopeless	0	1	2	3

(*For office coding: Total Score T*____ = ____ + ____ + ____)

Developed by Drs. Robert L. Spitzer, Janet B.W. Williams, Kurt Kroenke and colleagues, with an educational grant from Pfizer Inc. No permission required to reproduce, translate, display or distribute

New cut-off points of PHQ-9 and its variants, in Costa Rica: a nationwide observational study. https://doi.org/10.1038/s41598-023-41560-0
armando_gonzalez@usal.es

Patient Health Quesionnaire-9 (PHQ-9). Versión costarricense.

Instrucciones: Durante las últimas 2 semanas, ¿con qué frecuencia ha sentido molestias por los siguientes problemas? Siendo (0) Para nada, (1) Varios días, (2) Más de la mitad de los días y (3) Casi todos los días.

Nº	Ítem	0	1	2	3
1	Poco interés o placer en hacer las cosas				
2	Sentirse con depresión, sin ánimo o sin esperanzas				
3	Problemas para iniciar o mantener el sueño o dormir demasiado				
4	Sentir cansancio o con poca energía				
5	Sentir poco apetito o comer en exceso				
6	Sentirse mal acerca de sí mismo o tener un sentimiento de fracaso o de abandono propio o de la familia				
7	Dificultad para concentrarse en diferentes actividades tales como leer el periódico o ver televisión				
8	Moverse o hablar tan despacio que otras personas lo han notado o bien, por el contrario, estar con tanta inquietud o intranquilidad, que se mueve mucho más de lo normal				
9	Pensamientos de deseo de muerte o que quisiera lastimarse de alguna manera				

Si ha marcado alguno de los problemas de este cuestionario, ¿hasta qué punto estos problemas le han creado dificultades para hacer su trabajo, ocuparse de la casa o relacionarse con los demás? Conteste con (0) Ninguna dificultad, (1) Un poco de dificultad, (2) Mucha dificultad y (3) Extremada dificultad.

Nº	Ítem	0	1	2	3
10	Si usted tuvo molestias por alguno de los problemas mencionados, ¿cuánta dificultad le causaron estos problemas para **hacer su trabajo**?				
11	Si usted tuvo molestias por alguno de los problemas mencionados, ¿cuánta dificultad le causaron estos problemas para **encargarse de las tareas del hogar**?				
12	Si usted tuvo molestias por alguno de los problemas mencionados, ¿cuánta dificultad le causaron estos problemas para **relacionarse con los demás**?				

Patient Health Quesionnaire-4 (PHQ-4). Versión costarricense.

Instrucciones: Durante las últimas 2 semanas, ¿con qué frecuencia ha sentido molestias por los siguientes problemas? Siendo (0) Para nada, (1) Varios días, (2) Más de la mitad de los días y (3) Casi todos los días.

Nº	Factor	Ítem	0	1	2	3
1	Depresión	Poco interés o placer en hacer las cosas				
2	Depresión	Sentirse con depresión, sin ánimo o sin esperanzas				
3	Ansiedad	Sentir nervios, angustia o mucha tensión				
4	Ansiedad	No poder dejar de preocuparse o no poder controlar la preocupación				

Corrección: Cuanto más elevada sea la puntuación, más probabilidad existe de que haya un trastorno depresivo o de ansiedad. Se corrigen ambos test por separado. Puntuaciones mayores a 3 se consideran como caso.

To score each test, you must add up your scores.

To score the PHQ-9: You must add the responses of the 9 items of the PHQ-9.
To score the PHQ-8: You must add the responses of the first 8 items of the PHQ-9.
To score the PHQ-4: You must add the responses of the 4 items of the PHQ-4.
To score the PHQ-2: You must add the responses of the first 2 items of the PHQ-9.

Once the sum is done, you should consult the attached correction table. To know the score, you should direct your attention to the part of the table corresponding to the test you want to correct. Within that test, you should select the score obtained from the y-axis. Finally, you should select the population to which the subject to whom the test was applied belongs: men, women, or general population. The score of the original version is added for comparison purposes.

To help interpret the cut-off points, the following example is offered:

The PHQ-9 test has been applied to a man, obtaining a score of 15 points after adding his first 9 items. When inspecting the table, we should look at the score of 15 on the vertical axis and in the PHQ-9 column, specifically in the man's column. It can be seen that these coordinates indicate a value of 3. This value of 3 corresponds to "Moderate Depression

---

Para corregir cada test debe realizar una suma de sus puntuaciones.

Para corregir el PHQ-9: Debe sumar las respuestas de los 9 ítems del PHQ-9.
Para corregir el PHQ-8: Debe sumar las respuestas de los 8 primeros ítems del PHQ-9.
Para corregir el PHQ-4: Debe sumar las respuestas de los 4 ítems del PHQ-4.
Para corregir el PHQ-2: Debe sumar las respuestas de los 2 primeros ítems del PHQ-9.

Una vez realizada la suma deberá consultar la tabla de corrección adjunta. Para conocer la puntuación deberá dirigir su atención a la parte de la tabla correspondiente al test que desea corregir. Dentro de ese test deberá seleccionar la puntuación obtenida del eje de las íes. Por último deberá seleccionar la población a la que se adscribe el sujeto a quien se le aplicó el test: varones, mujeres o población general. Se añade la puntuación de la versión original a efectos de comparación.

Para ayudar a la interpretación de los puntos de corte se ofrece el siguiente ejemplo:

Se le ha aplicado el test PHQ-9 a un varón, obteniendo una puntuación de 15 puntos tras sumar sus 9 primeros ítems. Al inspeccionar la tabla, debemos fijarnos en la puntuación de 15 en el eje vertical y en la columna PHQ-9, específicamente en la columna de varón. Puede apreciarse que estas coordenadas indican un valor de 3. Este valor de 3 corresponde a "Depresión moderada".

New cut-off points of PHQ-9 and its variants, in Costa Rica: a nationwide observational study. https://doi.org/10.1038/s41598-023-41560-0
armando_gonzalez@usal.es

Scoring table. Tabla de corrección.

Visualization of the cut-off points of the PHQ test versions. Visualización de los puntos de corte de las versiones de los test PHQ.

	Test															
	PHQ-2				PHQ-4				PHQ-8				PHQ-9			
Puntuación	Original	General	Varón	Mujer	Original	General	Varón	Mujer	Original	General	Varón	Mujer	Original	General	Varón	Mujer
0	1	1	1	1	1	1	1	1	1	1	1	1	1	1	1	1
1	1	1	1	1	1	1	1	1	1	1	1	1	1	1	1	1
2	1	1	1	1	1	1	2	1	1	1	1	1	1	1	1	1
3	2	2	2	2	2	2	2	2	1	1	1	1	1	1	1	1
4	2	2	2	2	2	2	2	2	1	1	2	1	1	1	1	1
5	2	2	2	2	2	2	3	2	2	1	2	1	2	1	1	1
6	2	2	2	2	3	3	3	3	2	2	2	1	2	2	2	1
7					3	3	3	3	2	2	2	2	2	2	2	2
8					3	3	5	3	2	2	2	2	2	2	2	2
9					5	5	5	5	2	2	3	2	2	2	2	2
10					5	5	5	5	3	2	3	2	3	2	2	2
11					5	5	5	5	3	3	3	2	3	3	3	2
12					5	5	5	5	3	3	3	3	3	3	3	3
13									3	3	3	3	3	3	3	3
14									3	3	4	3	3	3	3	3
15									4	3	4	3	4	3	3	3
16									4	4	4	3	4	4	4	3
17									4	4	4	4	4	4	4	4
18									4	4	4	4	4	4	4	4
19									4	4	5	4	4	4	4	4
20									5	4	5	4	5	4	4	4
21									5	5	5	4	5	5	5	4
22									5	5	5	5	5	5	5	5
23									5	5	5	5	5	5	5	5
24									5	5	5	5	5	5	5	5
25													5	5	5	5
26													5	5	5	5
27													5	5	5	5

Note. For the PHQ-2 test, the value 1 refers to the Absence of major depressive disorder and 2 to its presence. For the rest of the tests 1: Minimal Depression; 2: Mild Depression; 3: Moderate Depression; 4: Moderately Severe Depression and 5: Severe Depression. The PHQ-4 can be analyzed as the depression-anxiety continuum or by its separate items grouping them by depression (first 2 items) and anxiety (last 2 items).

Original: (*Original*) scores as they appear in the original version of the tests. General: (*General*) scores for the Costa Rican population without considering their sex. Varón: (*Male)* scores for the Costa Rican population considered male. Mujer: (*Female*) scores for the Costa Rican population considered female.

Nota. Para el test PHQ-2, el valor 1 se refiere a Ausencia de trastorno depresivo mayor y el 2 a su presencia. Para el resto de test, 1: Depresión Mínima; 2: Depresión Leve; 3: Depresión Moderada; 4: Depresión Moderadamente severa y 5: Depresión Severa. El PHQ-4 puede analizarse como el continuo depresión-ansiedad o por sus ítems por separado agrupándolos por depresión (2 primeros ítems) y ansiedad (2 últimos ítems).
